# Screening tests for aphasia in patients with stroke: a systematic review

**DOI:** 10.1007/s00415-016-8170-8

**Published:** 2016-06-03

**Authors:** Hanane El Hachioui, Evy G. Visch-Brink, Lonneke M. L. de Lau, Mieke W. M. E. van de Sandt-Koenderman, Femke Nouwens, Peter J. Koudstaal, Diederik W. J. Dippel

**Affiliations:** 1 Department of Neurology, Erasmus MC University Medical Center, Room EE 2291, P.O. Box 2040, 3000 CA Rotterdam, The Netherlands; 2 Department of Neurology, Slotervaart Medical Center, Amsterdam, The Netherlands; 3 Rijndam Rehabilitation Institute, Rotterdam, The Netherlands; 4 Department of Rehabilitation Medicine and Physical Therapy, Erasmus MC University Medical Center, Rotterdam, The Netherlands

**Keywords:** Cerebrovascular disease/stroke, Infarction, Intracerebral hemorrhage, Diagnostic test assessment, Aphasia, Screening test

## Abstract

Aphasia has a large impact on the quality of life and adds significantly to the costs of stroke care. Early recognition of aphasia in stroke patients is important for prognostication and well-timed treatment planning. We aimed to identify available screening tests for differentiating between aphasic and non-aphasic stroke patients, and to evaluate test accuracy, reliability, and feasibility. We searched PubMed, EMbase, Web of Science, and PsycINFO for published studies on screening tests aimed at assessing aphasia in stroke patients. The reference lists of the selected articles were scanned, and several experts were contacted to detect additional references. Of each screening test, we estimated the sensitivity, specificity, likelihood ratio of a positive test, likelihood ratio of a negative test, and diagnostic odds ratio (DOR), and rated the degree of bias of the validation method. We included ten studies evaluating eight screening tests. There was a large variation across studies regarding sample size, patient characteristics, and reference tests used for validation. Many papers failed to report on the consecutiveness of patient inclusion, time between aphasia onset and administration of the screening test, and blinding. Of the three studies that were rated as having an intermediate or low risk of bias, the DOR was highest for the Language Screening Test and ScreeLing. Several screening tools for aphasia in stroke are available, but many tests have not been verified properly. Methodologically sound validation studies of aphasia screening tests are needed to determine their usefulness in clinical practice.

## Introduction

For people aged 65 years or more, the worldwide prevalence of stroke ranges from 46 to 73 per 1000 persons [1]. This number is likely to increase in the coming years due to aging of the population. Approximately 30 % of stroke survivors have aphasia in the acute phase of stroke [2], a condition affecting daily communication and thus quality of life. Aphasia adds significantly to the costs of patient care after stroke due to a longer hospital stay [3], and patients with aphasia are more frequently discharged to a rehabilitation center than those without [4]. The initial severity of aphasia is an important factor determining the prognosis of patients with aphasia due to stroke [5, 6]. It has repeatedly been suggested that the treatment of aphasia should be initiated as soon as possible after stroke, although consistent evidence for a beneficial effect of early language therapy has not been published yet [7].

Altogether, it is pivotal that the presence and severity of aphasia are adequately evaluated in patients who suffered a stroke. A large number of diagnostic instruments are available to examine the type and degree of aphasia. As many of these diagnostic test batteries are fairly demanding and time-consuming, they may be too cumbersome for stroke patients in the acute phase. Given that the aphasia characteristics are generally instable shortly after stroke and can change rapidly, extensive testing may be a waste of time and resources. In addition, a speech and language therapist (SLT) is not always sufficiently available in the first days after stroke to obtain a detailed linguistic profile. Hence, a short and simple screening test, easy to administer by various disciplines, is essential for referring patients for additional assessment and adequate language therapy. Furthermore, advice regarding communication may be better personalized using results from screening tests.

The aim of this review was to identify available screening tests for differentiating between aphasic and non-aphasic patients after stroke, and to evaluate the accuracy, reliability, and feasibility of those tests.

## Methods

### Search strategy

We searched PubMed, EMbase, Web of Science, and PsycINFO for published studies on screening tests aimed at assessing the presence and/or severity of aphasia in patients who suffered an ischemic or hemorrhagic stroke. The following search string was used for NLM Pubmed-Medline and was adapted for the other databases:

(cerebrovascular disorders[mesh:noexp] OR brain ischemia[mesh] OR intracranial embolism and thrombosis[mesh] OR intracranial hemorrhages[mesh] OR stroke[mesh:noexp] OR vertebral artery dissection[mesh:noexp] OR stroke*[tw] OR poststroke*[tw] OR cva[tw] OR cvas[tw] OR cerebrovasc*[tw] OR cerebral vasc*[tw] OR ((cerebr*[tw] OR intracerebr*[tw] OR cerebell*[tw] OR brain*[tw] OR vertebrobasilar*[tw] OR intracran*[tw]) AND (infarct*[tw] OR ischem*[tw] OR ischaem*[tw] OR hemorrh*[tw] OR haemorrh*[tw] OR hematom*[tw] OR haematom*[tw] OR thrombos*[tw] OR thrombot*[tw] OR thromboembol*[tw] OR thrombol*[tw] OR apoplex*[tw] OR emboli*[tw] OR bleed*[tw]))) AND (aphas*[tw] OR logastheni*[tw] OR logagnos*[tw] OR logamnes*[tw] OR alogi*[tw] OR anepia*[tw] OR dysphasi*[tw] OR lichtheim*[tw]) AND (test[tw] OR tests[tw] OR testing*[tw] OR screen*[tw] OR tool*[tw] OR instrument*[tw] OR assessment*[tw]) AND (accura*[tw] OR sensitiv*[tw] OR specificit*[tw] OR psychometr*[tw] OR psychometr*[tw] OR predictive value*[tw]). We applied no search limits. The reference lists of the selected articles were checked, and experts in the field of aphasia research were contacted to detect additional published studies. The initial search was carried out in March 2012 and updated in May 2015 with search in Pubmed.

### Selection of studies

Eligible for inclusion were full-text articles, written in Dutch, English, French, German or Spanish, on cohort or cross-sectional studies of stroke patients who underwent a screening test to detect aphasia. A screening test was defined as a diagnostic test designed to assess the presence and/or severity of aphasia, requiring a short turnaround time that is at most 15 min. Studies evaluating patients with aphasia due to other causes than stroke or with an unspecified etiology were not included. We also excluded studies in which test scores of aphasic stroke patients were compared with those from healthy controls instead of stroke patients without aphasia, as we specifically aimed to evaluate screening tests suitable for use in clinical practice.

Articles had to report the results of the screening test for aphasia as well as those from a reference test or gold standard. Data should be described in such a way that the sensitivity and specificity of the screening test could be calculated. If sensitivity and specificity were given without reporting the original data, the authors of the paper were contacted. In the case, authors were not able to provide the requested data; the study was excluded from this review.

First, titles and abstracts of the retrieved studies were checked, and obviously, irrelevant articles were excluded. If a decision could not be made based on the information in the title and abstract, then the full-text article was checked for the above-mentioned in- and exclusion criteria.

### Data extraction

From the selected studies, we recorded the clinical characteristics of the patient sample (age, sex, stroke type, and number of patients with and without aphasia). The following features of the validation method were collected: consecutiveness of patient inclusion, the type of reference test that was used, and blinding of the test assessors. All estimates of test accuracy reported in the studies had to be based on the exact numbers of patients and were recalculated to check for errors and non-verification (that is whether only patients who could be assessed with the reference as well as with the screening test were included and reported which indicates selection bias). We collected the following data on the screening tests: the language in which the validation study was conducted, subtests, score range, time needed for administration, and type of patients for which the test was initially developed, and reported suitability for bedside use.

### Data analysis

We expressed the results of the validation studies of each screening test in 2 × 2 tables and estimated the sensitivity, specificity, likelihood ratio of a positive test (LR+), and the likelihood ratio of a negative test (LR−). Sensitivity was estimated by the number of aphasic patients who were correctly classified with the screening test divided by the total number of patients with aphasia. Specificity was estimated by the number of patients without aphasia who were correctly classified divided by the total number of patients without aphasia. LR+ was estimated by the sensitivity divided by 1-specificity. LR− was estimated by dividing 1-sensitivity by the specificity [8]. The diagnostic odds ratio (DOR) was used as a single measure of test accuracy and was calculated by dividing the LR+ by the LR− [9].

We evaluated the methodological quality of the selected studies by scoring three items: consecutiveness of patient inclusion, representativeness of the patient sample, and blinding. Consecutive patient inclusion is essential to eliminate selection bias and to ensure that the full range of aphasia types and severities is represented in the patient sample. Furthermore, the patient sample should be representative for the general stroke population, since this is the population in which the screening test will be used. Blinding is of importance to minimize expectation bias. The assessor of the screening test should not be aware of the results of the reference test, and vice versa [8].

The score assigned for the representativeness of the patient sample in the validation study (‘0’ not representative or not reported, ‘1’ fairly representative or partially not reported, or ‘2’ very representative) was based on the size of the cohort, available data on stroke type, and mean age and sex of the patient sample. Consecutiveness was scored as either ‘0’ (no consecutive inclusion or consecutiveness not reported) or ‘2’ (consecutive inclusion of patients). The degree of blinding was rated as ‘0’ (when assessment was not blinded or blinding was not reported on), ‘1’ (in the case of blinding for the screening test only, or blinding without further specification), or ‘2’ (in the case of blinding for both the reference and the screening test).

Finally, we assigned a score for the risk of bias based on the three above-mentioned items. A total score of ≤2 was classified as high risk of bias, a total score of 3 or 4 as intermediate risk of bias, and a total score of ≥5 as low risk of bias.

## Results

**Fig. 1:**
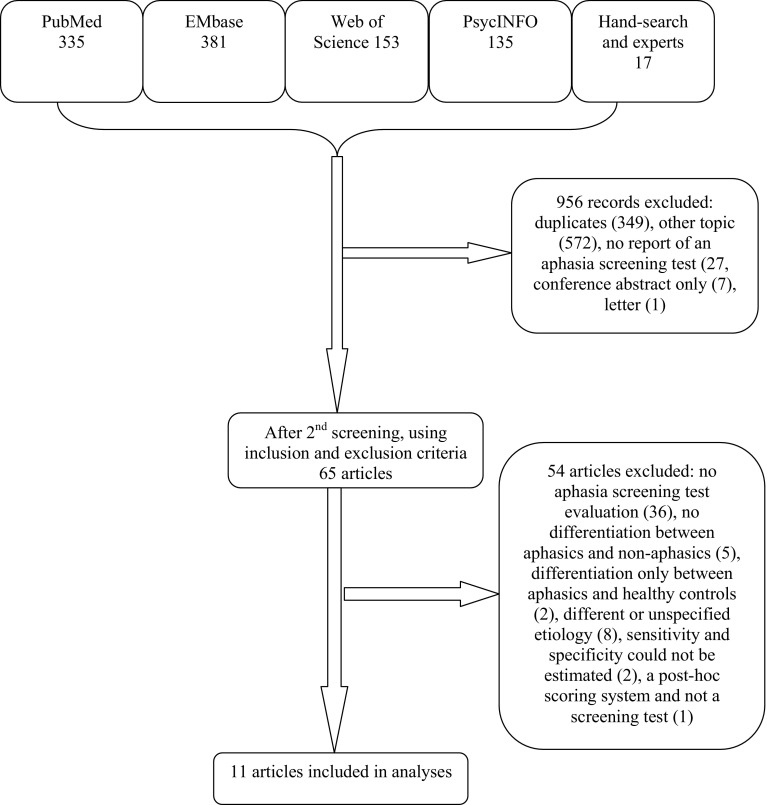
Flowchart of the search results

The electronic search resulted in 1004 records. We identified 13 additional articles after hand-searching the reference lists and another 4 by asking experts in the field. After screening all titles and abstracts, 956 records were excluded (Fig. 1). Sixty-five full-text articles were assessed for eligibility, of which 14 were selected. There were no articles excluded because of the administration time of the test. In three articles, the sensitivity and specificity were reported, but the exact numbers of evaluated patients were lacking. After contacting the publication authors, we retrieved the data for one of these papers. The other two studies [10, 11] were excluded, as the requested data were not available. One article reported on the aphasia item of the Scandinavian Stroke Scale (SSS). This study did not meet the inclusion criteria, since the SSS is a post hoc scoring system and not a screening test. Eventually, we included 11 articles, including 1 review [12]. In total, eight screening tests for aphasia were evaluated.

### Included studies

Table 1 shows the characteristics of the patient samples of the ten included validation studies (the table does not contain the review article [12]) ordered alphabetically by screening test. One paper reported on the validation of two screening tests [13], a full version and a short version of the same test, and two tests were evaluated in more than one study [13–18]. Sample sizes ranged from 37 [19] to 194 [16] patients. Only two studies provided details concerning the type of stroke (i.e., ischemic versus hemorrhagic) [18, 20]. In two papers, information on age and sex of the patient sample was lacking [14, 15], and another three evaluated a rather young cohort (i.e., mean age of 54 [13, 18] and 55 [17] years). In one study, the screening test was validated in the chronic stage [17], and in three studies, the time since stroke onset was not reported [13, 21, 22].

**Table 1:** Characteristics of the study cohorts of the validation studies

Study	Screening test	*N*	Stroke type^a^ (*n*/*n*)	Age (year) (mean)	Male sex *n* (%)	Time since onset (days)
Al-Khawaja 1996 [13]	FAST	50	n.r.^b^	54	32 (64)	n.r.
Enderby 1987 [14]	FAST	50	n.r.	n.r.	n.r.	1–36
O’Neill 1990 [15]	FAST	54	n.r.	n.r.	n.r.	1
Flamand-Roze 2011 [21]	LAST	102	n.r.	62	52 (51)	n.r.
Choi 2015 [18]	MAST*	60	41/19	54	47 (78)	2–8
Kostalova 2008 [16]	MAST	194	n.r.	68–71^c^	97 (50)	1–46
Romero 2012 [17]	MAST	58	10/19	55	32 (55)	277^d^
Doesborgh 2003 [20]	ScreeLing	63	54/9	62	43 (68)	2–11
Al-Khawaja 1996 [13]	SST	50	n.r.^b^	54	32 (64)	n.r.
Kim 2011 [22]	SVF	53	27/n.r.	66	36 (68)	n.r.
Thommessen 1999 [19]	UAS	37	n.r.	76	15 (41)	3–8

*FAST* Frenchay Aphasia Screening Test, *LAST* Language Screening Test, MAST* Mobile Aphasia Screening Test, *MAST* Mississippi Aphasia Screening Test, *SST* Sheffield Screening Test for acquired language disorders, *SVF* semantic verbal fluency, *UAS* Ullevaal Aphasia Screening Test, *n.r.* not reported

^a^ *n* (ischemic stroke)/*n* (hemorrhagic stroke)

^b^8 patients with traumatic brain injury were included in the study

^c^Median

^d^Mean

### Screening tests included in the review

We included validation studies for nine screening tools: the full and the short version of the Frenchay Aphasia Screening Test (FAST) [13–15], Language Screening Test (LAST) [21], Mississippi Aphasia Screening Test (MAST) [16, 17, 23], the mobile aphasia screening test (also abbreviated as MAST) [18], ScreeLing [20], Sheffield Screening Test for Acquired Language Disorders (SST) [13], Semantic Verbal Fluency (SVF) [22], and Ullevaal Aphasia Screening test (UAS) [19]. Characteristics of the screening tests, including language, subtests, score range, administration time, type of patients, the test was originally designed for, and applicability as a bedside screening tool are given in Table 2. Two of the validation studies were conducted in English, two were conducted in Korean, one in Norwegian, one in French, one in Czech, one in Spanish, and one in Dutch [13–15, 23, 24]. All tests can be administered within 15 min and most of them are judged to be suitable for bedside use. The SVF [22] was originally designed for patients with dementia [24]. The SST [13] and the MAST [23] were not developed specifically for stroke patients, but to assess language deficits in general. The mobile aphasia screening test is a tablet application based on the Korean version of the FAST and explicitly designed with no tool requirements so to be used for patients in remote locations easily [18].

**Table 2:** Characteristics of the screening tests

Screening test	Language in which the study was conducted	Subtests	Score range	Administration time	Designed for	Bedside
FAST	English	Full form: comprehension; expression; reading; writing	0–30 (full)	10 min	Stroke	Yes
		Short form: comprehension; expression	0–20 (short)	3 min		
LAST	French	Naming; repetition; automatic speech; picture recognition; executing verbal orders	0–15	2 min	Stroke	Yes
MAST*	Korean	Expression; comprehension	0–20	3 min	Stroke	n.r.
MAST	Czech and Spanish	Naming; automatic speech; repetition; following instructions; yes/no responses; writing/spelling; object recognition; reading and executing instructions; verbal fluency	0–100	5–10 min	Severely impaired language/communication	Yes
ScreeLing	Dutch	Semantics; phonology; syntax	0–72	15 min	Stroke	Yes
SST	English	Receptive skills; expressive skills	0–20	3–5 min	Suspected language disorders	Yes
SVF	Korean	Semantic fluency: animals	n.a.	i. 60 s	Dementia	n.r.
				ii. 30 s		
UAS	Norwegian	Expression; comprehension; repetition; reading; word strings; writing; free communication	n.r.	5–15 min	Stroke	n.r.

*n.a.* not applicable

### Methodological quality of the validation studies

Table 3 provides information on methodological features for each validation study, including reference test used, test assessors and blinding of test assessors, and consecutiveness of patient inclusion. In more than half of the studies, patients were not included consecutively [13, 16–18, 21, 22]; and for one study, this information was missing [14]. The diagnostic test that was applied as the gold standard varied from an informal evaluation by an SLT to extensive aphasia test batteries. In most studies, the reference diagnosis was made by an SLT [13–16, 19]; in two studies, this information was not reported [21, 22] or not exactly specified [17]. The screening tests were carried out by various disciplines. Most studies did not provide information on the time interval between the assessment of the reference test and the screening test [13–15, 20–22], as was the case with respect to the order in which the assessments were conducted [13, 14, 16, 17, 20, 22].

**Table 3:** Methodological features of the validation studies

Study	Screening test	Reference	Assessor of reference test	Assessor of screening test	Cut-off for screening test	Blinding^a^	Consecutive inclusion
Al-Khawaja 1996 [13]	FAST	SLT	SLT	Non-specialist, n.f.s.	17^b^; 16^c^; 15^d^ (short)	n.r.	No
Enderby 1987 [14]	FAST	SLT, FCP, sS	SLT	n.r.	23 (full); 14 (short)	n.r.	n.r.
O’Neill 1990 [15]	FAST	sS, BDAE	SLT	Physician	25	n.r.	Yes
Flamand-Roze 2011 [21]	LAST	BDAE	n.r	SLT, nurse, neurologist, or student	15	Yes, n.f.s.	No
Choi 2015 [18]	MAST*	Physiatrist	Physiatrist	Research assistant, test scored by SLT	16^j^; 14^k^	n.r.	No
Kostalova 2008 [16]	MAST	SLT	SLT	Neurology resident and student	93^e^; 96^f^; 98^g^	n.r.	No
Romero 2012 [17]	MAST	BDAE, TT	Clinical expert, n.f.s.	Clinical expert, SLT, neurologist	90	No	No
Doesborgh 2003 [20]	ScreeLing	TT, exp.	Neurologist, linguist	n.r.	66	Yes, 3	Yes
Al-Khawaja 1996 [13]	SST	SLT	SLT	Non-specialist, n.f.s.	17^h^; 16^i^; 15^d^	n.r.	No
Kim 2011 [22]	SVF	STAND	n.r.	n.r.	60 s; 7/30 s; 6	n.r.	No
Thommessen 1999 [19]	UAS	SLT, parts of NGA	SLT	Nurse	n.r.	Yes, 2	Yes

*SLT* speech and language therapist, *FCP* functional communication profile, *sS* short Schuell, *BDAE* Boston Diagnostic Aphasia Examination, *TT* Token Test, *exp.* expert assessment, *NGA* Norsk grunntest for afasi (Norwegian Basic Aphasia Assessment), *TAND* Screening Test for Aphasia and Neurologic-Communication Disorders, *n.f.s.* not further specified, *n.r.* not reported

^a^Blinding: 1 for reference test only, 2 for screening test only, 3 for reference and screening test

^b^For age ≤59 years

^c^For age 60–70 years

^d^For age ≥71 years

^e^For age ≤60 years

^f^For age 61–70 years

^g^Basic and secondary education

^h^Academic education, age ≥60 years

^i^Academic education, age <60 years

^j^For age ≤64 years

^k^For age >64 years

One study lacked blinding [17], in one study, blinding was reported to be secured, but it was not specified how [21], and seven studies did not describe whether or not test assessors were blinded [13–16, 18, 22]. Three studies reported on cut-off scores for the screening test indicating the presence or the absence of aphasia, which were stratified for age [13, 16, 18], and in one study, no cut-off score was reported [19]. In three studies [13, 15, 16], the cut-off value for the screening test was based on the previous studies comparing subjects with aphasia and healthy control persons.

Table 4 shows the diagnostic properties of the identified aphasia screening tests (sensitivity, specificity, LR+, LR−, and DOR). In all studies, every patient was reported to be assessed with the reference test as well as with the screening test. Four studies included a larger group of patients with aphasia than without aphasia [13, 16, 21, 22], and two included groups of equal sample size [17, 18]. In five studies, the DOR was infinite, because either LR− was nil or LR+ was infinite [13, 14, 17, 21].

**Table 4:** Diagnostic properties of the validation studies

Study	Screening test	Aphasia (*n*)	No aphasia (*n*)	Aphasia correctly classified (*n*)	No aphasia correctly classified (*n*)	Sensitivity (%)	Specificity (%)	Non-verified (*n*)	LR+	LR−	DOR (95 % CI)
Al-Khawaja 1996 [13]	FAST	45	5	39	4	87	80	0	4.4	0.16	27.5 (2.6–289.5)
Enderby 1987 [14]	FAST, full	20	30	20	23	100	77	0	4.4	0.00	∞
Enderby 1987 [14]	FAST, short	20	30	20	27	100	90	0	10	0.00	∞
O’Neill 1990 [15]	FAST	23	31	22	19	96	61	0	2.5	0.07	35.7 (4.2–300.5)
Flamand-Roze 2011 [21]	LAST	52	50	51	50	98	100	0	∞	0.02	∞
Choi 2015 [18]	MAST*	30	30	27	22	90	73	0	3.37	0.14	24.7 (5.9–104)
Kostalova 2008 [16]	MAST	149	45	143	40	96	89	0	8.7	0.04	217.5 (63.1–749.7)
Romero 2012 [17]	MAST	29	29	26	29	90	100	0	∞	0.10	∞
Doesborgh 2003 [20]	ScreeLing	14	49	12	47	86	96	0	21.5	0.15	143.3 (18.3–1124.3)
Al-Khawaja 1996 [13]	SST	38	4	35	4	92	80	0	∞	0.08	∞
Kim 2011 [22]	SVF, 60 s	27	26	23	22	85	85	0	5.7	0.18	31.7 (7.0–142.5)
Kim 2011 [22]	SVF, 30 s	27	26	23	23	85	88	0	7.1	0.17	41.8 (8.4–208.0)
Thommessen 1999 [19]	UAS	8	29	6	26	75	90	0	7.5	0.28	26.8 (3.6–197.5)

*LR+* Likelihood Ratio of a Positive Test, *LR*− Likelihood Ratio of a Negative Test, *DOR* diagnostic odds ratio

^a^8 patients have traumatic brain injury

^b^Data on the 42 stroke patients could separately be extracted

In Table 5, the estimated degree of bias is given based on scores for blinding of test assessors, consecutiveness of inclusion, and representativeness of the patient sample. Seven studies were judged as having a high risk of bias, two as having an intermediate risk of bias, and in one study, the risk of bias was judged low. Four screening tools seemed to perform very good (Table 4), with sensitivity and specificity of 100 and 90 %, respectively (short version of FAST [14]), 98 and 100 % (LAST [21]), 86 and 96 % (ScreeLing [20]), and 90 and 100 % in one ([17]) and 96 and 89 % in another study [16] (MAST). However, the validation studies for the FAST short version and both validation studies for the MAST were considered as having a high risk of bias. Of the three studies with an intermediate or low risk of bias, the calculated DOR was highest for the LAST [21] and Screeling [20].

**Table 5:** Risk of bias in evaluated validation studies

Study	Screening test	Score for blinding^a^	Score for consecutiveness^b^	Score for representativeness^c^	Risk of bias^d^
Al-Khawaja 1996 [13]	FAST	0	0	1	High
Enderby 1987 [14]	FAST	0	0	0	High
O’Neill 1990 [15]	FAST	0	2	0	High
Flamand-Roze 2011 [21]	LAST	1	0	2	Intermediate
Choi 2015 [18]	MAST*	0	0	2	High
Kostalova 2008 [16]	MAST	0	0	2	High
Romero 2012 [17]	MAST	0	0	1	High
Doesborgh 2003 [20]	ScreeLing	2	2	2	Low
Al-Khawaja 1996 [13]	SST	0	0	1	High
Kim 2011 [22]	SVF	0	0	2	High
Thommessen 1999 [19]	UAS	1	2	1	Intermediate

^a^0: assessment was not blinded or blinding was not reported on, 1: blinding for the screening test only, or blinding without further specification, 2: blinding for both the reference and the screening test

^b^0: no consecutive inclusion or consecutiveness not reported, 2: consecutive inclusion of patients

^c^Based on the size of the cohort, available data on stroke type, and mean age and sex of the study population, 0: not representative or not reported, 1: fairly representative or partially not reported, 2: very representative

^d^Total score ≤2: high, total score ≥3 and ≤4: intermediate, total score ≥5: low

## Discussion

Given the impact of aphasia on the quality of life, rehabilitation after stroke, and the costs of stroke care [25], it is of great importance that aphasia in stroke patients is immediately recognized, allowing for adequate referral and treatment as soon as possible. Hence, it is crucial to have a brief and easy screening test for aphasia that may be administered by SLTs as well as other health professionals shortly after aphasia onset and is also suited for ill stroke patients for whom an extensive test battery is too demanding. A simple screening tool for aphasia may also be of use for research purposes, to identify patients with aphasia in stroke trials.

In this systematic review, we evaluated ten studies reporting on the validation of eight screening tests for aphasia after stroke, with emphasis on the methodological quality of the validation study. Nearly, all included screening tools usually reflect the approach taken in the traditional aphasia test batteries that assess language modalities, such as spontaneous speech, auditory and written comprehension, reading and writing in addition to naming and repetition, except the ScreeLing and the SVF. The ScreeLing comprises tasks directly aimed at the basic linguistic components (semantics, phonology, and syntax). The SVF addresses semantic verbal fluency only. Although it is not always explicitly mentioned in the test descriptions, all tests are suitable to be administered at bedside, a requirement for the use in the acute stage.

Several issues have to be taken into account when appraising studies that claim to validate a screening test against a reference test [8]. Clearly, the patient sample of the validation study should be representative for the population in which the screening test will be applied. This means that a screening tool for aphasia due to stroke should be verified in a cohort representative for the general stroke population. For this reason, we only included validation studies performed on stroke patients with and without aphasia, and excluded studies investigating test performance by examining aphasic stroke patients and healthy controls. We attempted to assign a score for representativeness to each included study based on the available information on patient characteristics. Unfortunately, data on age and sex of the patient sample were not reported for all studies. Furthermore, in more than half of the validation studies, patients were not included consecutively, or this information was missing. Consecutive inclusion increases the likelihood that the full spectrum of aphasia severity is represented in the study cohort and minimizes the risk of selection bias. The 1:1 ratio of patients with and without aphasia in some of the validation studies [17, 18, 22], however, suggests that the patients were not recruited consecutively but rather selected. One study that reported consecutive inclusion only enrolled patients already suspected to have aphasia, resulting in a study cohort containing a majority (i.e., 90 %) of stroke patients with aphasia [13]. In all the studies, the number of non-verified patients was nil, which indicates that selection bias may have been present to some extent. It is possible that only patients who were able to undergo the screening test as well as the reference test were enrolled, while patients for whom the burden of the reference test (which is likely to be more time-consuming and more difficult) was too high were not included. In addition, the administration of the reference test should not be restricted to patients in whom the screening test was positive, to avoid workup bias. In each study included in this review, all patients were reported to be assessed both with the screening test and the test used as the gold standard.

For many of the screening tools, the cut-off value below or above which the test result is considered abnormal (i.e., the patient is diagnosed as having aphasia) was derived from studies performed in stroke patients with aphasia and healthy control subjects, while cut-off values based on a general stroke population are preferred. Finally, the assessor of the screening test should be blind for the result of the reference test and the other way around. Many of the evaluated studies did not report whether or not blinding was secured, making it difficult to estimate the risk of expectation bias. Altogether, most of the validation studies had serious methodological limitations, thus hampering firm conclusions about utility of the aphasia screening tools for clinical practice.

Of the four studies with an intermediate or low risk of bias, the LAST [21] and Screeling [20] seem to have the best diagnostic properties. An advantage of the LAST is the short administration time. The ScreeLing, a measure for the patients’ functioning in the main linguistic levels semantics, phonology, and syntax, gives more detailed information for language treatment. It is notable that the SVF, a very short screening test that was initially developed for use in patients with dementia, also performs quite reasonably as a screening test for aphasia in stroke patients [22].

Besides the screening tools evaluated in this review, there are several well-known screening tests for aphasia that are widely used in clinical practice. For the Acute Aphasia Screening Protocol [26], the Aachen Aphasia Bedside Test [27], and the Bedside Western Aphasia Battery [28], strikingly, we were unable to find any peer-reviewed articles in which these tests were validated in stroke patients with and without aphasia. The Token Test [29] is one of the first recommended screening tests for the detection of aphasia in patients with neurological damage and, therefore, exists in a lot of variants [30–32]. However, although this test is generally considered very useful in clinical practice, it could not be included, because the etiology of aphasia was too diverse or unspecified in the validation studies for this test. Finally, general stroke scales quantifying stroke severity in the acute stage contain specific subparts for speech and language, such as the NIH Stroke Scale (NIHSS) [33], the Canadian Neurological Scale (CNS) [34], and the European Stroke Scale (ESS) [35]. These standardized scales are often used in clinical practice to identify stroke patients with aphasia, but have not been systematically validated as such.

In conclusion, several screening tools for aphasia in stroke are available, but many tests have not been verified in a proper way. Future studies should focus on a better validation of the available aphasia screening tests in large stroke populations. The design should include a reliable reference diagnosis, a consecutive inclusion of stroke patients to make them representative of a general stroke population, a secured blinding of the assessments, details on the numbers of aphasics and non-aphasics correctly classified, and a good description of the subtests of the screening test, to eliminate the risk of bias as much as possible.
